# Euglycemic DKA (euDKA) as a presentation of COVID‐19

**DOI:** 10.1002/ccr3.3540

**Published:** 2020-11-20

**Authors:** Bhagwan Dass, Andrew Beck, Cody Holmes, Glenville Morton

**Affiliations:** ^1^ Eglin Air Force Base Eglin AFB FL USA

**Keywords:** COVID‐19, diabetes, diabetes mellitus, diabetes type 2, diabetic ketoacidosis, euglycemia, type I diabetes mellitus

## Abstract

COVID‐19 in the setting of SGLT2 inhibitor use may precipitate euglycemic DKA separate from known acute viral illness and dehydration precipitants. There should be consideration of proactive discontinuation of these medications in these patients.

## Graphical abstract

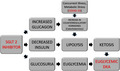

## 1 INTRODUCTION

Euglycemic DKA (euDKA) is a rare but serious side effect that has been found to be associated with SGLT2 inhibitors in patients with type 2 diabetes. Given their undisputed cardiovascular and renal benefits, these medications are common in patients with type 2 diabetes. With the emergence of COVID‐19, we have learned that patients with type 2 diabetes are predisposed to more serious course of illness and complications from COVID‐19 infection. As we learn more daily about COVID‐19, we are also finding that the virus itself may affect vital organs involved in glucose metabolism. We present a case euglycemic DKA in a type 2 diabetic patient on an SGLT‐2 inhibitor likely precipitated by COVID‐19 infection. We suspect that COVID‐19 itself, separate from known acute viral illness and dehydration precipitants, led to euDKA and worsening of underlying diabetes as she required insulin upon discharge for blood glucose control. We also raise the question of whether these medications should be held or discontinued in patients who are under investigation or test positive for COVID‐19 in not only the inpatient, but proactively in the outpatient setting.

Diabetic ketoacidosis (DKA) is a medical emergency characterized by hyperglycemia, metabolic acidosis, and ketosis. EuDKA differs from typical DKA in that it often presents with serious metabolic acidosis but only mild‐to‐moderate glucose elevation (<200 mg/dL).^1^ Known precipitants for euDKA include severe acute illness, dehydration, extreme physical activity, surgery, low carbohydrate intake, fasting, excessive alcohol intake, and SGLT2 inhibitors.^2^

Diabetes is associated with an increased risk of severe COVID‐19 with both higher morbidity and mortality rates in patients with diabetes mellitus.^3^, ^4^ SARS‐CoV‐2 utilizes the ACE‐2 receptor for viral entry, which is expressed in several organs, and may have diabetogenic effects beyond the well‐recognized stress response associated with severe illness. The virus may cause alterations of glucose metabolism at the tissue level directly and indirectly that could complicate existing diabetes.^5^ COVID‐19 may alter the pathophysiology of preexisting diabetes or worsen it with associated complications such as ketoacidosis.

## 2 CASE PRESENTATION

**FIGURE 1:**
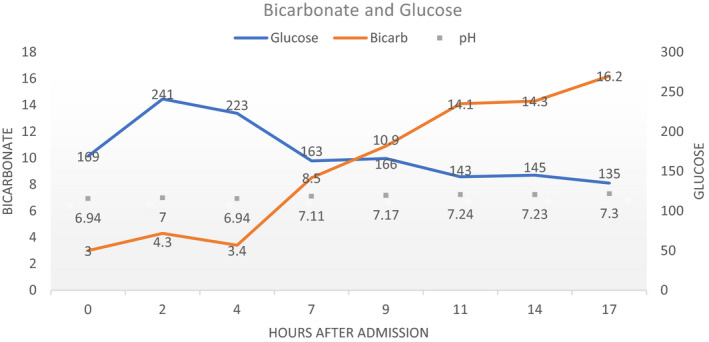
Laboratory values over the initial course of hospitalization

A 59‐year‐old female with history of documented type 2 diabetes on empagliflozin, sitagliptin, and metformin presented with 9 days of progressively worsening shortness of breath, low‐grade fevers, and fatigue. She was seen 2 days prior in the emergency department and had an elevated glucose of 198, normal CO2 of 22 (normal 20‐31) on basic metabolic panel and was found to have bilateral infiltrates on chest x‐ray. She was diagnosed with community acquired pneumonia and discharged on doxycycline. On representation to the ED <48 hours later, she presented with tachypnea and tachycardia and was found to have a profound metabolic acidosis with significant respiratory compensation with an associated nongap acidosis as seen on her initial ABG (pH of 6.94, PaCO2 of 13, PaO2 of 99 and a HCO3 of 3) (Figure 1). On serum analysis, her lactate was 0.9, her glucose 154, confirmed bicarb of <10, serum osmolality of 346, an elevated anion gap of 30, beta‐hydroxybutyrate of 95. Her urinalysis showed 3+ glucose and 2+ ketones. She had a negative UDS and salicylate levels were normal. She was found to have a positive COVID‐19 test and was admitted for euglycemic DKA (euDKA). She was started on an insulin drip and IV fluid and had resolution over the next 2 days. After resolution of DKA, the patient was continued on her sitagliptin and metformin, but empagliflozin was discontinued given the association of SGLT2 inhibitors with euDKA. She was also started on 20 units of insulin glargine nightly which was continued upon discharge.

## 3 DISCUSSION

We performed a literature search of PubMed using a combination of the words “euglycemic diabetic ketoacidosis,” “COVID‐19” with “SGLT2 inhibitors.” To our knowledge, this is the first case report in the literature that documents DKA with normal glucose levels in the setting of COVID‐19 and SGLT2 inhibitor use in type 2 diabetes (there is a case report in type 1).

Diabetic ketoacidosis (DKA) is a medical emergency characterized by hyperglycemia (blood sugar > 250 mg/dL), metabolic acidosis (arterial pH < 7.3 and serum bicarbonate < 18 mEq/L), and ketosis—euglycemic DKA includes blood glucose levels <200 mg/dL, arterial pH < 7.3, anion gap > 12 mEq/L, HCO3− <15 mEq/L and the presence of ketones in blood and urine.^1^ EuDKA was first described as a discrete entity by Munro et al in 1973.^6^ EuDKA differs from typical DKA in that it often presents with serious metabolic acidosis but only mild‐to‐moderate glucose and anion gap elevation. Diagnosis should be confirmed with the direct measurement of the beta‐hydroxybutyrate level in blood and arterial blood pH.^7^ Known precipitants for euDKA include severe acute illness, dehydration, extreme physical activity, surgery, low carbohydrate intake, fasting, excessive alcohol intake, and SGLT2 inhibitors.^2^

The increased risk of euDKA associated with SGLT2 inhibitors is well known. In 2015, the FDA released safety warnings about the risk of euDKA associated with SGLT2 inhibitors.^8^ The development of euDKA with SGLT2 inhibitors is thought to involve decreased insulin production and increase in secretion of glucagon. Increase in glucagon levels is multifactorial including both direct and indirect mechanisms. It is increased directly via effects on SGLT2 expressed in the glucagon‐secreting alpha pancreatic cells. SGLT2 inhibitors also increase glucose excretion leading to relative lower levels of insulin and low ratio of insulin to glucagon. The relative lack of insulin stimulates the production of free fatty acids and ketone bodies and a shift from glucose to fat metabolism causing ketoacidosis.^9^

Ketone production may be further stimulated in the setting of SGLT2 inhibitors by the lowering of glucose reabsorption in the proximal tubules increasing glycosuria and possibly simulating starvation conditions.^10^ Ketones can be excreted in the urine as sodium salts and are essentially the equivalent to the loss of bicarbonate causing metabolic acidosis.^11^

**FIGURE 2:**
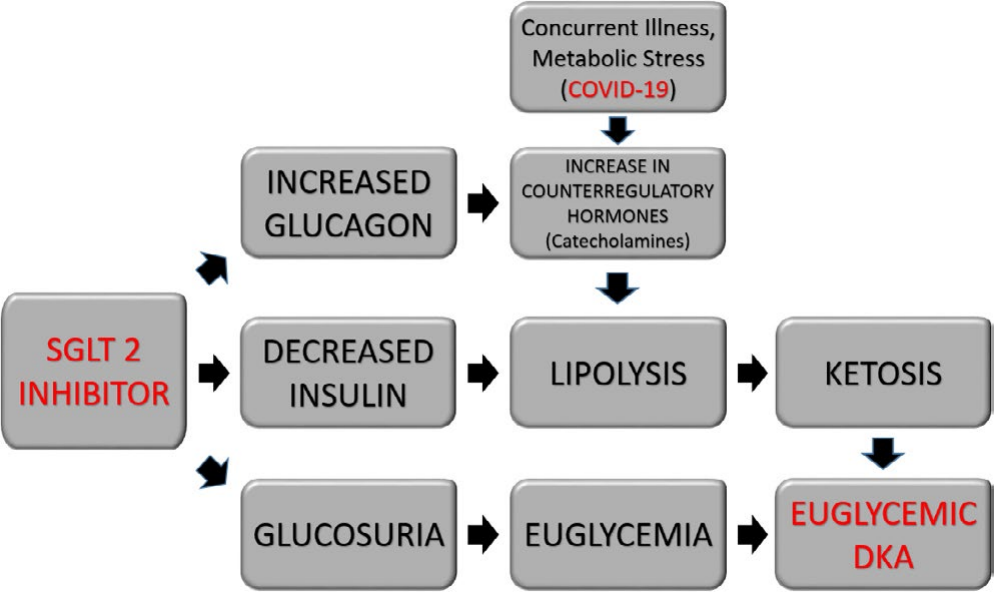
Role of SGLT 2 inhibitors and COVID‐19 in the development of euglycemic DKA

In addition to SGLT2 inhibitors, the COVID‐19 virus itself may be directly linked to the development of euDKA (Figure 2). Studies have shown that COVID‐19 utilizes the ACE‐2 receptor, which is expressed on human pancreatic beta cells, to gain entry to and infect human cells. Once inside the cell, an immune response is triggered which leads to the production of cytokines and chemokines resulting directly in cell death.^12^ A similar effect was seen in the similar SARS‐CoV‐1 coronavirus in which the virus also utilized the ACE2 receptor in the islet cells of the pancreas to gain entry leading to direct cellular destruction precipitating acute diabetes in a subset of patients.^5^ Acute infection with COVID‐19 could lead to further decrease in insulin production and predisposition to euDKA. Low pH in DKA also favors the entry and replication of the SARS‐CoV‐2, and development of DKA makes the disease course of COVID‐19 worse.^13^

In this case, the development of euDKA was likely multifactorial in the setting of COVID‐19 infection and SGLT2 inhibitor use and it is likely that COVID‐19 was an inciting factor for euDKA. Our patient who was on metformin and empagliflozin prior to COVID‐19 infection ended up being discharged on insulin most likely due to combination of COVID‐19 illness and impaired beta cell function in setting of SARS‐CoV‐2 Infection.

Physicians need to acquaint themselves with euDKA to promptly recognize and treat this medical emergency. This is particularly imperative in this pandemic of COVID‐19 and wide‐spread use of SGLT2 inhibitors given their proven cardiovascular and renal benefits. We believe the risk of SGLT2‐inhibitor‐associated euDKA can be reduced or prevented by the discontinuation of these medications during acute illness with COVID‐19.

As the majority of COVID‐19 patients with type 2 diabetes are managed as an outpatient with home quarantine, one possible option is to stop SGLT2 inhibitors or reduce their dose.

As research is evolving, there could be a potential role for dipeptidyl peptidase 4 (DPP4) inhibitors. DPP4 inhibitors might interfere with and modify viral binding and hypothetically reduce virulence. DPP4 inhibitors modulate inflammation and exert antifibrotic activity. These properties may be of potential use for halting progression to the hyperinflammatory state associated with severe COVID‐19.^14^ This interaction deserves further investigation and possibly DPP4 inhibitor trial during active COVID‐19 infection.

## CONFLICT OF INTEREST

None declared.

## AUTHOR CONTRIBUTIONS

BD, AB, and CH: wrote manuscript, revised, and approved manuscript. MG, AB, and CH: involved in patient management, revised manuscript, and approved manuscript.

## ETHICAL APPROVAL

Informed consent was obtained from the patient for the study and can be reproduced upon request. This case report conforms to recognized ethical standards.

## Data Availability

Data were obtained from the EMR at our facility and are reproducible upon request.
